# The association of α4β7 expression with HIV acquisition and disease progression in people who inject drugs and men who have sex with men: Case control studies

**DOI:** 10.1016/j.ebiom.2020.103102

**Published:** 2020-11-07

**Authors:** Alyssa R. Martin, Eshan U. Patel, Charles Kirby, Jacquie Astemborski, Gregory D. Kirk, Shruti H. Mehta, Kyle Marshall, Holly Janes, Ashley Clayton, Lawrence Corey, Scott M. Hammer, Magdalena E. Sobieszczyk, James Arthos, Claudia Cicala, Andrew D. Redd, Thomas C. Quinn

**Affiliations:** a Laboratory of Immunoregulation, Division of Intramural Research, National Institute of Allergy and Infectious Diseases, National Institutes of Health, Bethesda, MD, United States; b Department of Pathology, Johns Hopkins University School of Medicine, Baltimore, MD, United States; c Department of Epidemiology, Johns Hopkins Bloomberg School of Public Health, Baltimore, MD, United States; d Vaccine and Infectious Disease Division, Fred Hutchinson Cancer Research Center, Seattle, WA, United States; e Department of Medicine, Columbia University Medical Center, New York, NY, United States; f Department of Medicine, Johns Hopkins University School of Medicine, Baltimore, MD, United States

## Abstract

**Background:**

α4β7 is a gut-homing integrin heterodimer that can act as a non-essential binding molecule for HIV. A previous study in heterosexual African women found that individuals with higher proportions of α4β7 expressing CD4^+^ T cells were more likely to become infected with HIV, as well as present with faster disease progression. It is unknown if this phenomenon is also observed in men who have sex with men (MSM) or people who inject drugs (PWID).

**Methods:**

MSM and transgender women who seroconverted as part of the HVTN 505 HIV vaccine trial and PWID who seroconverted during the ALIVE cohort study were selected as cases and matched to HIV-uninfected controls from the same studies (1:1 and 1:3, respectively). Pre-seroconversion PBMC samples from cases and controls in both studies were examined by flow cytometry to measure levels of α4β7 expression on CD4^+^ T cells. Multivariable conditional logistic regression was used to compare α4β7 expression levels between cases and controls. A Kaplan-Meier curve was used to examine the association of α4β7 expression pre-seroconversion with HIV disease progression.

**Findings:**

In MSM and transgender women (*n* = 103 cases, 103 controls), there was no statistically significant difference in the levels of α4β7 expression on CD4^+^ T cells between cases and controls (adjusted odds ratio [adjOR] =1.10, 95% confidence interval [CI]=0.94,1.29; *p* = 0.246). Interestingly, in PWID (*n* = 49 cases, 143 controls), cases had significantly lower levels of α4β7 expression compared to their matched controls (adjOR = 0.80, 95% CI = 0.68, 0.93; *p* = 0.004). Among HIV-positive PWID (*n* = 47), there was no significant association in HIV disease progression in individuals above or below the median level of α4β7 expression (log-rank *p* = 0.84).

**Interpretation:**

In contrast to findings in heterosexual women, higher α4β7 expression does not predict HIV acquisition or disease progression in PWID or MSM.

**Funding:**

This study was supported in part by the Division of Intramural Research, National Institute of Allergy and Infectious Diseases (NIAID), National Institutes of Health. The study was also supported by extramural grants from NIAID T32AI102623 (E.U.P.), and UM1AI069470.

**Research in context**

**Evidence before this study**

The α4β7 integrin complex is found on the surface of T cells and targets the cells to the gut and other mucosal barriers. It can also act as non-essential binding molecule for human immunodeficiency virus (HIV). Studies in a non-human primate SIV infection model have shown that administration of antibodies against the α4β7 complex can be protective from HIV infection during viral challenge. In a recent detailed study of two groups of heterosexual African women at high risk of HIV infection, higher pre-infection levels of α4β7 expressing CD4^+^ T cells were found to be associated with increased susceptibility to infection, and also appeared to accelerate the rate of disease progression post-infection. We searched PubMed, MEDLINE, and Web of Science for English-language articles published before July 30, 2020, using the search terms “HIV”, “HIV acquisition”, or “HIV Disease Progression” and linked them with “α4β7” and “α4β7 integrin”. We found only one published reports of a study of the direct role of α4β7 expression on HIV acquisition and disease progression.

**Added value of this study**

In contrast to a previous report of increased risk of HIV acquisition and faster disease progression for heterosexual women with higher levels of α4β7 expression on their CD4^+^ T cells, this study found no association with higher expression of α4β7 and increased risk of HIV acquisition in men who have sex with men and transgender individuals, or disease progression in people who inject drugs. Interestingly, this study also found that in people who inject drugs, the opposite was true in that individuals who became infected with HIV had lower α4β7 expression on their CD4^+^ T cells prior to acquiring HIV.

**Implications of all the available evidence**

This report, taken with the previous study in heterosexual African women, suggests that the role of α4β7 expression on CD4^+^ T cells may depend on the mode of transmission, the biological sex of the individual, racial background, or some combination of all these components. Further research is warranted to clarify this relationship.

Alt-text: Unlabelled box

## 1 Introduction

The α4β7 integrin complex is a heterodimer found on the surface of lymphocytes that when activated targets these cells to the gut and other mucosal barriers [1]. It has also been shown that α4β7 can act as non-essential binding molecule for human immunodeficiency virus (HIV), and expression of α4β7 can increase the susceptibility of CD4^+^ T cells to HIV-1 infection *in vitro* [2]. It has been hypothesized that α4β7 expression by CD4^+^ T cells could have an important role in HIV acquisition, as well as pathogenesis by helping to disseminate infected T cells throughout the body, particularly to the gut and other mucosal sites [2,3]. Depletion of CD4^+^ T cells in the gut-associated lymphoid tissue (GALT) during HIV infection has been observed in several studies regardless of transmission route, and this depletion may be related to the high levels of α4β7^hi^ cells that reside at mucosal sites like the gut [4], [5], [6].

*In vivo* studies in a non-human primate (NHP) SIV infection model have shown that administration of a monoclonal antibody against the α4β7 complex can be protective from HIV infection during mucosal challenge, and decrease the loss of CD4^+^ T cells in GALT [7]. In addition to a possible role in HIV acquisition and pathogenesis, a NHP SIV study also identified a potential role for α4β7 in the context of treated chronic infection [8]. In particular, this study found that SIV-infected macaques who were treated with antiretroviral therapy (ART) and an α4β7 blocking antibody appeared to control the rebound of their virus when the ART and antibody were removed [8]. However, this study was not confirmed in subsequent NHP studies, nor in one study in ART-suppressed people living with HIV (PLWH) [9], [10], [11], [12].

In a recent study, in two groups of women in South Africa and Kenya at high risk for heterosexual acquisition of HIV infection, higher pre-infection levels of α4β7 expressing CD4^+^ T cells were found to be associated with increased susceptibility to infection, and also exhibited an accelerated rate of disease progression [13]. However to date, these associations have not been studied in men, nor in the context of other routes of transmission.

Therefore, two case-control studies were performed in groups of men who have sex with men (MSM) and people who infect drugs (PWID), respectively, to examine if higher levels of α4β7 expression were associated with increased HIV acquisition, and if expression was associated with faster disease progression in PWID.

## 2 Methods

### 2.1 Study population: HIV Vaccine Trials Network 505

The HIV Vaccine Trials Network (HVTN) 505 study procedures have been described previously [14]. Briefly, HIV-uninfected MSM and transgender women were randomized to receive either a recombinant DNA plasmid vaccine at months 0, 1, and 2 followed by a recombinant adenoviral vector vaccine injection at month 6, or placebo injections [14]. PBMC samples were collected at enrollment as a baseline (pre-injection), month 2.5, month 7 (1 month post-final injection), month 12, 24, and 36 (for protocol versions 1 and 2 that included month 36). Participants who acquired HIV during the study with available samples prior to acquiring HIV were selected from both arms of the trial and individually matched 1:1 to controls without replacement. Only cases that had sufficient stored PBMCs (>1 vial) at an HIV-negative visit within 1 year of diagnosis were eligible for inclusion. Cases were matched to controls on enrollment time within 3 months and if PBMC samples were available from the same visit during study duration (incidence density sampling). Selection of samples was prioritized for those individuals with previously collected immunogenicity data available, which was enriched for controls in the per-protocol cohort [14]. Participants (both cases and controls) that had HIV-uninfected samples available at two time points before and after vaccination or placebo administration were also examined for changes in α4β7 expression by the vaccine regimen. For case-control analysis, later time points (closest time point pre-acquisition) were used for participants with multiple samples tested. Patient demographics and characteristics collected at baseline and associated with risk of HIV infection, including a previously published baseline HIV risk score, were also collected [14].

### 2.2 Study population: AIDS linked to the intravenous experience

PBMC samples were also obtained from participants in the AIDS Linked to the IntraVenous Experience (ALIVE) study. The original study procedures have been previously described [15]. Briefly, ALIVE has recruited PWID or people who have a history of injection drug use regardless of HIV status and follows them longitudinally with semi-annual visits. The study participants are provided with counseling, testing and access to HIV and drug addiction care. Patient demographics and characteristics associated with risk of HIV infection are collected at each study visit and participants are tested for HCV and HIV seroconversion in a prospective manner. HIV seroconverters who had pre-seroconversion samples available were individually matched to uninfected controls with available samples without replacement. Samples were selected at a 1:3 case:control ratio and matched on age (±10 years), race, active injection drug use in the past 6 months and year of sampling (±3 years). 51 potential cases were identified; two cases had zero matched controls and so were excluded, one case had two matched controls available and was included in analysis. Three control samples, each matched to a different case, were of insufficient quality and thus were not included in analysis. Data on CD4^+^ T cell counts were also collected following HIV seroconversion, allowing investigation of the potential effect of α4β7 expression on HIV disease progression.

All participants provided written informed consent, and both studies were approved by all pertinent institutional review boards.

### 2.3 Quantification of α4β7 and other cellular factors

Flow cytometry was used to measure the expression of cell markers (LSR II, BD Biosciences). Quantification of α4β7 levels was performed by gating β7^+^CD45RA^−^ within the population of CD4^+^ T cells to identify α4β7^hi^ populations based on previously published data that demonstrated that αEβ7, the only other form of β7 found on human T cells, is rarely found in the blood [2,13,16,17]. PBMCs were stained with the following combination of antibodies from BD Biosciences: APC-H7-CD3 (560176), PerCP-Cy5.5-CD45RA (563429), BV421-CCR5 (562576), PE-Integrin β7 (555945), BB515-CD25 (564467), PE-Cy7-CD27 (560609), PE-CF594-HLA-DR (562304), BUV395-CD8 (563795), BUV496-CD38 (564657); and the following antibodies from Biolegend: BV605-CCR6 (353420), AlexaFluor 700-CD4 (344622), BV510-CCR7 (353232), AlexaFluor 647-CD127 (351318) (Supplemental Figure S1).

### 2.4 Statistical analyses

Based on previous data [13], a power calculation was used to determine the optimal number of samples for each case-control setup to maximize likelihood of observing an association of α4β7 expression with HIV acquisition if one existed in these populations. To have a 95% chance of detecting the same odds ratio of 1.17 with 80% power, 97 cases and 97 controls would be needed in a 1:1 setup using the previous study's median level of α4β7^hi^CD4^+^ cells expression of 8.7%; alternatively, 70 cases and 70 controls would be needed using a less conservative estimate of 7%. Based on this approach, a 1:3 case-control setup would require 59 cases and 177 controls at 8.7%, or 47 cases and 141 controls at 7%. These calculations did not account for adjustments for covariates.

Characteristics of the study populations were examined by case status using Fishers exact tests and Wilcoxon rank-sum tests for categorical and continuous variables, respectively. For both case-control analyses, univariable and multivariable conditional logistic regression were used to examine differences in the percent of α4β7^hi^CD4^+^ cells between cases and controls; odds ratios (OR) and corresponding 95% confidence intervals (CI) are reported. The multivariable model for the HVTN study of MSM and transgender women included baseline variables previously known to be risk factors for HIV (i.e., age [continuous], race/ethnicity, behavioral risk score), as well the per-protocol treatment status from the parent study (vaccine vs. placebo). Treatment status was included in the multivariable analysis because a trend toward increased HIV infection risk was previously shown in the vaccine group [14]. The multivariable model for PWID included adjustment for known sociodemographic and behavioral risk factors of HIV in this population (sex, cocaine use in the past six months, and number of sexual partners in the past six months) that had not already been accounted for via matching [18]. Age was also included as a continuous term in the multivariable model despite matching on age group to avoid potential residual confounding. All variables in the PWID model were ascertained from the sample visit.

Although PWID in the ALIVE study have scheduled semi-annual visits, PBMC samples were not always available from visits close to HIV seroconversion. Thus, a post-hoc sensitivity analysis was performed to assess for potential effect modification of the association between α4β7 and HIV seroconversion by time of α4β7 testing prior to HIV seroconversion (<1.5 and ≥1.5 years before HIV seroconversion). We added an interaction term between α4β7 and the time of α4β7 testing prior to HIV seroconversion to the main univariable and multivariable models to test for heterogeneity using a Wald test, and provide stratified effect estimates by time before HIV seroconversion.

A secondary analysis was used to examine changes in α4β7 expression before and after placebo or vaccine administration in the HVTN study population. Wilcoxon signed-rank tests were run to determine whether the percent of α4β7^hi^CD4^+^ cells was significantly different pre- and post-intervention.

To examine the role of α4β7 expression on disease progression, HIV seroconverters in the ALIVE study populations were divided at the median according to their percent CD4^+^ T cells expressing high levels of α4β7 pre-seroconversion, and the two groups (below vs. at and above the median) were analyzed using a Kaplan-Meier curve for time to CD4 count <200 cells/µL and a log-rank test.

Data analysis was performed in Stata/MP version 15.1 (College Station, Texas, USA). All *p*-values are two-tailed, and a *p*-value less than 0.05 was used to indicate statistical significance.

## 3 Results

**Fig. 1:**
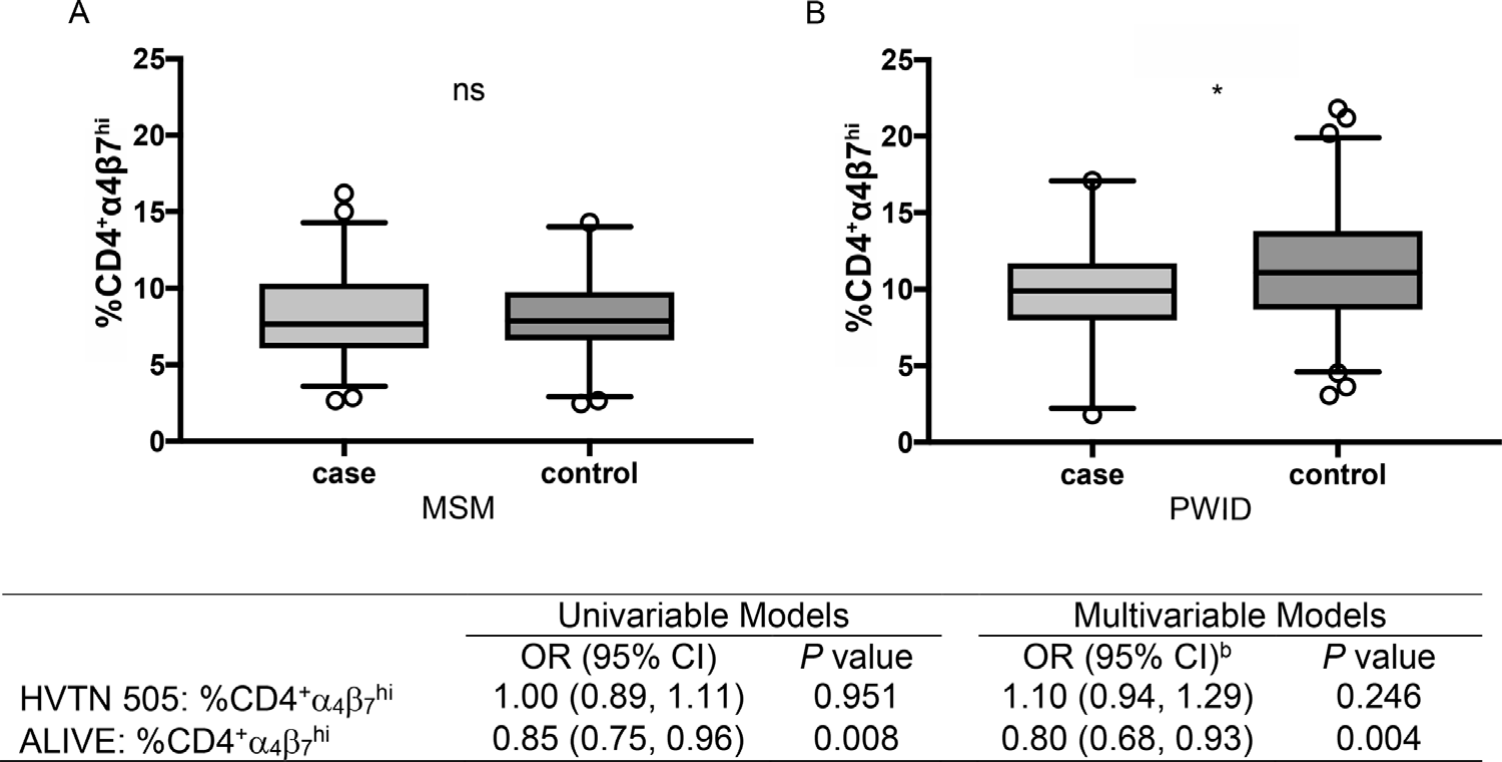
Association between α_4_β_7_ expression and HIV seroconversion. Differences measured in percent of α4β7^hi^CD4^+^ T cells between cases and controls in MSM (A) and PWID (B). Statistical analysis was done using conditional logistic regression between matched cases and controls. (ns-nonsignificant; *-*p* < 0.05, by conditional logistic regression). PWID in the ALIVE study were matched on age group, race, injection drug use in the past six months. For MSM and transgender women in the HVTN 505 vaccine trial, the multivariable model was adjusted for age, race/ethnicity, behavioral risk score, and vaccine group (per protocol). For PWID in the ALIVE study, the multivariable model was adjusted for age (continuous), sex, injection cocaine use in the past six months, and number of sexual partners in the past six months.

PBMC samples were selected from pre-seroconversion time points from MSM and transgender women who acquired HIV during the HVTN 505 vaccine trial (cases; *n* = 103), and matched to controls (*n* = 103) who remained uninfected on a 1:1 basis. Controls were not matched to cases according to any potential risk factors. The majority of PBMC samples were from time points that were <9 months before the estimated date of acquisition, and all samples were within one year of estimated HIV acquisition (Table 1). Compared to controls, cases were younger (*p* = 0.004), had significantly higher behavioral risk scores (*p* < 0.001), and had a greater proportion of individuals in the placebo group of the parent study based on intention-to-treat and per-protocol exposures (*p* < 0.05; Table 1). No statistically significant association between percent of α4β7^hi^CD4^+^ cells and HIV seroconversion was observed in univariable analysis (OR, 1.00 [95% CI: 0.89, 1.11]; *p* = 0.951) or in multivariable analysis (adjOR, 1.10 [95% CI: 0.94, 1.29]) that included adjustment for age, race/ethnicity, the behavioral risk score, and per-protocol intervention status (Fig. 1).

**Table 1:** Characteristics of men who have sex with men (MSM) and transgender women from the HVTN 505 vaccine trial study population.

Characteristic	Controls (*n* = 103)	Cases (*n* = 103)	*P* value
Age (years), median (IQR)	32 (25,40)	27 (23, 33)	**0.004**
Age group (years)			0.090
20-24	25 (24.3%)	35 (34.0%)	
25-29	18 (17.5%)	28 (27.2%)	
30-34	20 (19.4%)	18 (17.5%)	
35-39	14 (13.6%)	6 (5.8%)	
40-44	14 (13.6%)	8 (7.8%)	
≥45	12 (11.7%)	8 (7.8%)	
Gender Identity, %			0.748
Male	102 (99.0%)	100 (97.1%)	
Female	1 (1.0%)	1 (1.0%)	
Transgender Female	0 (0.0%)	2 (1.9%)	
Race/ethnicity, %			0.415
Non-Hispanic White	70 (70.0%)	64 (62.1%)	
Non-Hispanic Black	18 (17.5%)	27 (26.2%)	
Hispanic	8 (7.8%)	8 (7.8%)	
Non-Hispanic other race/multiracial	7 (6.8%)	4 (3.9%)	
Behavioral risk score [14]			**<0.001**
Low risk (score = 0)	35 (34.0%)	19 (18.4%)	
Low-medium risk (score = 0.46)	29 (28.2%)	18 (17.5%)	
Medium-high risk (score = 0.54)	20 (19.4%)	19 (18.4%)	
High risk (score = 1)	19 (18.4%)	47 (45.6%)	
Intention-to-treat trial arm			**0.016**
Placebo	33 (32.0%)	51 (49.5%)	
Vaccine	70 (68.0%)	52 (50.5%)	
Per-protocol trial arm			**<0.001**
Placebo	11 (10.7%)	34 (33.0%)	
Vaccine	92 (89.3%)	69 (67.0%)	
Time before HIV infection (days), median (range)		142 (17-361)	–
Time before HIV infection, %			–
≤3 months	–	34 (33.0%)	
3-9 months	–	49 (47.6%)	
>9 months	–	20 (19.4%)	

Note: Data are sample sizes and corresponding column percentages, unless otherwise indicated. *P* values were calculated from Fisher's exact tests, and do not account for the matched design of the study.

**Fig. 2:**
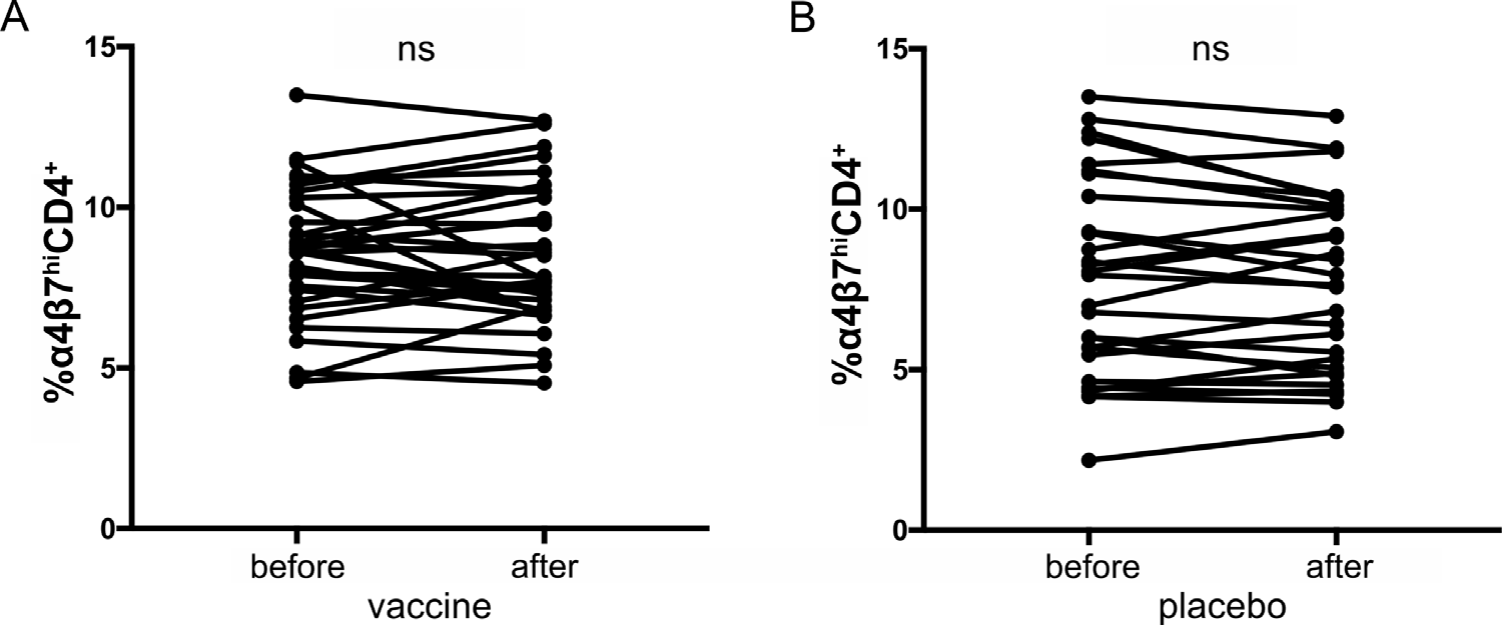
Levels of α4β7^hi^CD4^+^ T cells before and after vaccine or placebo administration. Proportion of α4β7^hi^CD4^+^ T cells in individuals from the HVTN 505 vaccine study before and after administration of the vaccine (A) or placebo (B) is shown. No significant changes were observed in either arm. (n.s, nonsignificant, by Wilcoxon signed-rank test)

In a separate analysis of cases and controls in the HVTN 505 vaccine trial that had two samples available before and after vaccination (*n* = 33) or placebo (*n* = 29), α4β7 expression was examined to account for a potential difference in infection risk that may have been mediated by vaccine administration. No difference in the levels of α4β7^hi^CD4^+^ cells was measured in vaccine or placebo recipients (Fig. 2).

To examine the role of α4β7 expression on the likelihood of HIV acquisition in PWID, pre-seroconversion PBMC samples from cases (*n* = 49) were selected from the ALIVE cohort and compared to matched controls (*n* = 143) in a 1:3 ratio. For cases, the median time between pre-seroconversion sample collection at which α4β7 expression was measured and the estimated date of seroconversion was 1.4 years (interquartile range [IQR], 0.2–10.1). Cases and controls were matched on age (±10 years), race, active injection drug use in the past six months and year of sampling (±3 years). No statistically significant difference was observed by age, sex, race, or measured behavioral risk factors between cases and controls (Table 2). Surprisingly, cases were found to have significantly lower levels of pre-seroconversion α4β7^hi^ CD4^+^ T cells when compared to controls (OR, 0.85 [95% CI = 0.75, 0.96]; *p* = 0.008) in univariable analysis, as well as in the multivariable model that additionally adjusted for age, sex, injection cocaine use in the past six months, and number of sexual partners in the past six months (adjOR, 0.80 [95% CI: 0.68, 0.93]; *p* = 0.004; Fig. 1).

**Table 2:** Characteristics of people who inject drugs (PWID) from the AIDS Linked to the IntraVenous Experience (ALIVE) study.

Characteristic	No. Participants (%)		*P* value
	Controls (*n* = 143)	Cases (*n* = 49)	
Age (years), median (IQR)	36 (32-42)	34 (31-39)	0.155
Age group (years)			0.516
20-24	9 (6.3%)	3 (6.1%)	
25-29	17 (11.9%)	9 (18.4%)	
30-34	37 (25.9%)	17 (34.7%)	
35-39	37 (25.9%)	9 (18.4%)	
40-44	25 (17.5%)	5 (10.2%)	
≥45	18 (12.6%)	6 (12.2%)	
Sex			0.865
Male	91 (63.6%)	32 (65.3%)	
Female	52 (36.4%)	17 (34.7%)	
Race			1.000
Black	135 (94.4%)	46 (93.9%)	
Non-black	8 (5.6%)	3 (6.1%)	
Injection drug use in past 6 months			1.000
No	35 (24.5%)	12 (24.5%)	
Yes	108 (75.5%)	37 (75.5%)	
Cocaine injection in past 6 months			0.122
No	61 (42.7%)	14 (28.6%)	
Yes	78 (54.5%)	32 (65.3%)	
*Missing*	4 (2.8%)	3 (6.1%)	
Heroin injection in past 6 months			0.522
No	64 (44.8%)	22 (44.9%)	
Yes	75 (52.4%)	24 (49.0%)	
*Missing*	4 (2.8%)	3 (6.1%)	
Frequency of injection drug use in past 6 months			0.736
None	35 (24.5%)	12 (24.5%)	
<Daily	50 (35.0%)	16 (32.7%)	
≥Daily	54 (37.8%)	18 (36.7%)	
*Missing*	4 (2.8%)	3 (6.1%)	
Number of sex partners in past 6 months			0.610
0	17 (11.9%)	3 (6.1%)	
1	68 (47.6%)	29 (59.2%)	
2	21 (14.7%)	7 (14.3%)	
≥3	29 (20.3%)	7 (14.3%)	
*Missing*	8 (5.6%)	3 (6.1%)	
Anti-HCV IgG status			0.547
Negative	14 (9.8%)	3 (6.1%)	
Positive	125 (87.4%)	46 (93.9%)	
*Missing*	4 (2.8%)	0 (0.0%)	
Time before HIV seroconversion (years), median (range)		1.4 (0.2-10.1)	-
Time before HIV seroconversion			-
<1.5 years	-	26 (53.1%)	
≥1.5 years	-	23 (46.9%)	

Note: Data are sample sizes and corresponding column percentages, unless otherwise indicated. *P* values were calculated from Wilcoxon rank sum tests and Fisher's exact tests for continuous and categorical variables, respectively, and do not account for the matched design of the study.

In a post-hoc sensitivity analysis, cases were stratified by time prior to seroconversion at which pre-infection α4β7 expression was measured (sampled <1.5 or ≥1.5 years pre-seroconversion). There were 26 case-sets sampled <1.5 years pre-seroconversion and 23 case-sets sampled >1.5 years pre-seroconversion for α4β7 testing. In univariable analysis, participants sampled within 1.5 years of estimated seroconversion had a significant association between a lower percentage of α4β7^hi^CD4^+^ T cells and risk of HIV acquisition (OR, 0.69 [95% CI: 0.56, 0.87]; *p* = 0.001), whereas those sampled outside of 1.5 years pre-seroconversion had no association (OR, 0.97 [95% CI: 0.84, 1.12]; *p* = 0.014) . This heterogeneity of effect remained significant in multivariable analysis (*p* = 0.007; Supplemental Table 1).

**Fig. 3:**
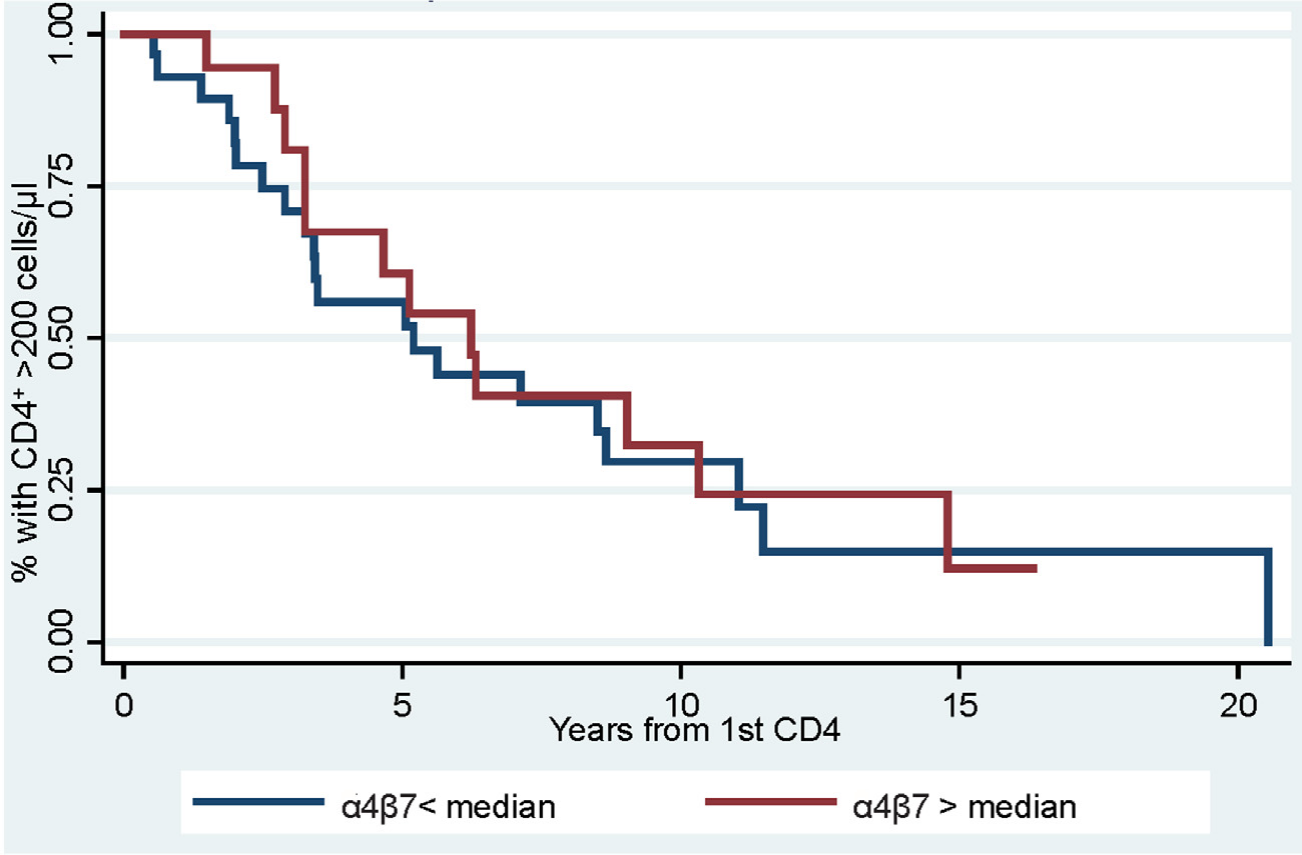
α_4_β_7_ expression and HIV disease progression in PWID. Kaplan-Meier analysis of time from estimated seroconversion to CD4^+^ T-cell count of <200 cells/µL of people below and at or above the median percentage of CD4+ T cells that expressed α4β7^hi^ levels is shown. There was no significant difference found (log-rank; *p* = 0.8449).

Lastly, to determine whether pre-infection α4β7 expression was associated with HIV disease progression, cases of PWID were examined for time to CD4^+^ T cell count of 200 cells/µL. This was possible because the majority of the cases from the ALIVE cohort were initially infected at a time antiretroviral therapy (ART) was either not available or only prescribed once a patient progressed to a CD4^+^ T cell count of 200 or less. In two patients, ART was administered before CD4^+^ T cell counts had declined below 200 cells/µL; therefore, these participants were excluded from the disease progression analysis. The remaining cases (*n* = 47) were separated by percent α4β7^hi^CD4^+^ T cells above and below the median measurement for this group (median = 10.5%). No difference in time to CD4^+^ T cell levels below 200 cells/µl was observed between these groups, suggesting that α4β7 expression was not associated with HIV disease progression (log-rank *p* = 0.8449; Fig. 3).

## 4 Discussion

In MSM and transgender women in the HVTN 505 HIV vaccine trial, there was no association between pre-infection α4β7 expression and HIV acquisition. Natural HIV disease progression data were not available for HVTN 505 participants who acquired HIV since they were rapidly prescribed ART. However, it was possible to examine the role of the vaccine regimen on possible changes in α4β7 expression. The samples used for this study were collected as part of a clinical trial testing an adenovirus serotype 5 (Ad5) vector-based vaccine. In this, and other studies using similar vaccine strategies, a trend or significant increase in infection risk was seen in recipients of the Ad5 vaccine [14,19]. Previous evidence has suggested that this apparent increase in susceptibility may be mediated, in part, by an expansion of α4β7^hi^ expressing adenovirus specific CD4^+^ T cells [20], however our data showed no difference in α4β7^hi^CD4^+^ T cells post-vaccination. Therefore, possible increased risk of HIV acquisition due to Ad5 vaccine vector exposure may not be associated with α4β7 expression, at least in this population of men and transgender women. It should also be noted that while transgender women were included in this analysis, the numbers were too small to make definitive statements on how α4β7 levels may affect HIV acquisition risk in this population.

It is believed that the role for α4β7 in HIV acquisition most likely occurs at either the mucosal barrier where viral infection is initiated, or in the early dissemination of the virus throughout the body, particularly to the gut. It is not therefore surprising that in PWID, where HIV is most often directly injected into the blood stream thereby bypassing the mucosal barrier, increased α4β7 expression was not associated with increased odds of HIV acquisition. However, the exact opposite effect was observed, with cases having significantly lower levels of α4β7 expression pre-seroconversion. It was also interesting that the association of HIV acquisition with lower α4β7 expression was only significant if the sample used for analysis was acquired within 1.5 years of HIV seroconversion. It is unclear what may be contributing to this phenomenon, but it is interesting to speculate that although the cases and controls were matched on active drug use in the past six months, there was a trend towards increased cocaine use in the cases versus the controls (*p* = 0.11). This is of interest because one characteristic of injection cocaine use is that in general it has been associated with higher HIV incidence presumably because of the requirement for more frequent injections [21].

The lack of association between α4β7 expression and disease progression in PWID was also in contrast to previously reported results. In the previous study in African women, HIV strains with a higher affinity for α4β7 binding were most likely to infect individuals with the highest levels of α4β7^hi^CD4^+^ T cells [13]. As α4β7 expression did not positively predict HIV acquisition in the PWID, the bias for high affinity α4β7 binding virus most likely did not exist in this population, which might explain why increased α4β7^hi^ levels were not associated with increased disease progression rates in this group. However it is known that CD4^+^ T cells in gut, which express high levels of α4β7, are preferentially targeted early in infection regardless of mode of transmission, and this may also explain the lack of association seen in this population of PWID.

There are several caveats to these analyses. In the case of the MSM population, men and transgender women were selected from an HIV vaccine clinical trial, and as stated above, the vaccine regimen may have affected their HIV risk. Although if there was an effect, the data presented here suggests it is independent of α4β7 expression. Lastly, in the PWID analysis, controls were matched to cases according to potential risk factors. This experimental setup, however, may have inhibited the further characterization of the potential link between lower α4β7 expression levels and increased HIV acquisition. Additionally, the PWID sample set had the limitation of sampling greater than a year before seroconversion for some participants. However, previous analysis of individuals from the ALIVE cohort demonstrated that the % CD4^+^ T cells that express high levels of α4β7 are extremely stable over several years [22]. Lastly, the previous report on α4β7 expression and HIV acquisition in African women controlled for HSV-2 status in their study, which was not available for these subjects [13].

Taken together, these data do not support a relationship between α4β7 expression and HIV acquisition and disease in PWID or MSM. While the data presented here suggests no role for increased α4β7 expression in HIV acquisition in the context of homosexual sex or intravenous drug use, the previous study using highly similar methods found an association between α4β7 and HIV in two distinct populations of African women [13]. Also in contrast to the findings in African women was the finding of no association of α4β7 expression and HIV disease progression. Yet, it is possible that these relationships are only found in individuals infected through heterosexual sex, and also may only be found in women and not in men. Further examination of α4β7 expression in both men and women exposed to HIV through heterosexual sex is warranted to clarify these issues.

## Data sharing statement

Data is available upon request (aredd2@jhmi.edu) and pending IRB approval.

## Contributions

Alyssa R. Martin-Writing; figure development; data collection, analysis and interpretation; designed study

Eshan U. Patel-Data analysis

Charles Kirby-Data collection

Jacquie Astemborski- Data collection and management; sample acquisition; study design

Gregory D. Kirk-Study design, sample acquisition

Shruti H. Mehta-Study design, sample acquisition

Kyle Marshall-Data management; sample acquisition; study design

Holly Janes-Data management; sample acquisition; study design

Ashley Clayton-Data management; sample acquisition

Lawrence Corey-Study design, sample acquisition

Scott M. Hammer-Study design, sample acquisition

Magdalena E. Sobieszczyk-Study design, sample acquisition

James Arthos-Study Design; technical assistance

Claudia Cicala-Study Design; technical assistance

Andrew D. Redd-Writing; data interpretation; designed study

Thomas C. Quinn-Project oversight; designed study

## Declaration of Competing Interest

Dr. Mehta reports personal fees from Gilead Sciences, outside the submitted work. No other conflicts of interest exist.

## Appendix Supplementary materials

Image, application 1

Image, application 2
