# Supplementary figures and images for: The association of α4β7 expression with HIV acquisition and disease progression in people who inject drugs and men who have sex with men: Case control studies
Source: eBioMedicine. 2020 Nov 7;62:103102. doi: 10.1016/j.ebiom.2020.103102 (PMC7658649; doi:10.1016/j.ebiom.2020.103102)

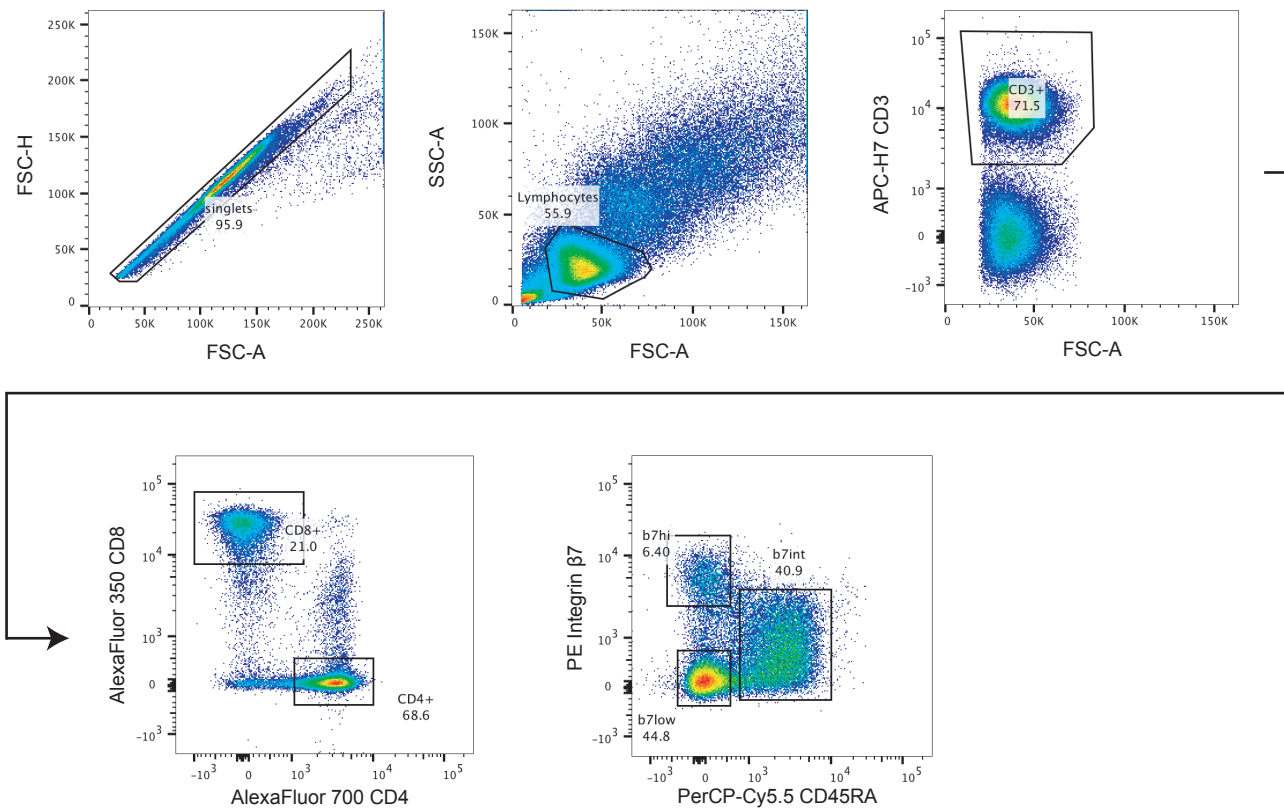

Supplementary Figure 1: Gating strategy used to determine CD4<sup>+</sup> B7<sup>hi</sup> expression.

Supplement: Supplementary file 1 [file mmc1.pdf]
