# Supplementary material for: The association of α4β7 expression with HIV acquisition and disease progression in people who inject drugs and men who have sex with men: Case control studies
Source: eBioMedicine. 2020 Nov 7;62:103102. doi: 10.1016/j.ebiom.2020.103102 (PMC7658649; doi:10.1016/j.ebiom.2020.103102)
Supplement: Supplementary file 2 [file mmc2.docx]

SUPPLEMENTAL MATERIAL

Supplemental Table 1. Association between α_4_β_7_^hi^ and HIV seroconversion among people who inject drugs stratified by sampling time before seroconversion.

| Measurement of %CD4^+^α_4_β_7_^hi^ | No.  Case-sets | Univariable^a^ | |  | Multivariable^a, b^ | | *P*_interaction_ |
| --- | --- | --- | --- | --- | --- | --- | --- |
|  |  | OR (95% CI) | P value |  | OR (95% CI) | P value |  |
| <1.5 years pre-seroconversion | 26 | 0.69 (0.56, 0.87) | 0.001 |  | 0.34 (0.15, 0.74) | 0.007 | 0.014 |
| ≥1.5 years pre-seroconversion | 23 | 0.97 (0.84, 1.12) | 0.661 |  | 0.92 (0.78, 1.09) | 0.334 | 0.007 |

^a^ PWID were matched on age group, race, injection drug use in the past 6 months.

^b^ The multivariable model was adjusted for age (continuous), sex, injection cocaine use in the past 6 months, and number of sexual partners in the past 6 months.
